# 

**Published:** 1898-06

**Authors:** 


### The Library of the Bristol Medico-Chirurgical Society.

*The following donations have been received since the publication of the list in March:*

*May 31st*, 1898.

| | |
|---|---|
| Joshua S. Beddome (1) ... ... ... ... ... ... ... | 4 volumes. |
| J. Paul Bush (2) ... ... ... ... ... ... ... ... | 4 ,, |
| Detroit Public Library (3) ... ... ... ... ... ... | 2 ,, |
| James Fawn & Son (4) ... ... ... ... ... ... | 38 ,, |
| L. M. Griffiths (5) ... ... ... ... ... ... ... ... | 1 volume. |
| Mrs. Henry Marshall (6) ... ... ... ... ... ... ... | 146 volumes. |
| Staff of Middlesex Hospital (7) ... ... ... ... ... | 1 volume. |
| J. E. Prichard, M.B., and Arthur W. Prichard (8) | 73 volumes. |
| W. U. Reber, M.D. (9) ... ... ... ... ... ... ... | 25 ,, |
| R. Shingleton Smith, M.D. (10) ... ... ... ... ... | 1 volume. |

Unbound periodicals have been received from Mr. J. Paul Bush, Dr. J. E. Prichard and Mr. Arthur W. Prichard. A framed picture has been presented by Mrs. Henry Marshall and unframed pictures have been received from Dr. J. E. Prichard and Mr. A. Ellis Wynter.

### TWENTY-EIGHTH LIST OF BOOKS.

The titles of books mentioned in previous lists are not repeated.

The figures in brackets refer to the figures after the names of the donors, and show by whom the volumes were presented. The books to which no such figures are attached have either been bought from the Library Fund or received through the *Journal.*

| | | | |
|---|---|---|---|
| **Abernethy, J.** | *Surgical Observations.* 2 parts | (6) | 1804-6 |
| **Adams, Sir W.** | *On Artificial Pupil* | (8) | 1819 |
| **Alison, W. P.** | *Outlines of Pathology and Practice of Medicine* | (8) | 1843 |
| **Allbutt, T. C.** [Ed.] | *A System of Medicine.* Vol. V. | | 1898 |
| **Anderson, I.** | *Yellow Fever in the West Indies* | | 1898 |
| **Ashton, T. J.** | *Diseases of the Rectum and Anus* | (6) 4th Ed. | 1863 |
| **Astruc, J.** | *De Morbis Venereis.* 2 vols. | (8) Editio Altera | 1740 |
| **Balfour, G. W.** | *Diseases of the Heart* | 3rd Ed. | 1898 |
| **Bannatyne, G. A.** | *Rheumatoid Arthritis* | 2nd Ed. | 1898 |
| **Barker, T. H.** | *On Malaria and Miasmata* | (8) | 1863 |
| **Barnes, R.** | *Diseases of Women* | (6) | 1873 |
| **Barth et H. Roger,** [J.-B.-P.] | *Traité pratique d'Auscultation* | (8) | 1841 |
| **Bastian, H. C.** | *Aphasia and other Speech Defects* | | 1898 |
| **Beard, J.** | *On Certain Problems of Vertebrate Embryology* | | 1896 |
| **Beasley, H.** | *Book of Prescriptions* | (4) | 1854 |
| **Beevor, C. E.** | *Diseases of the Nervous System* | | 1898 |
| **Bennet, J. H.** | *On Inflammation of the Uterus* | (6) 2nd Ed. | 1849 |
| **Birch, S. B.** | *Constipated Bowels* | (4) | 1861 |
| **Bird, G.** | *Natural Philosophy* | (6) 3rd Ed. | 1848 |
| **Bradshaw, W.** | *The Anatomy of Dyspepsia* | (4) | 1864 |
| **Brodie, T. G.** | *Essentials of Experimental Physiology* | | 1898 |
| **Brown, J.** | *Elementa Medicinæ.* 2 vols. in 1 | (8) | 1784 |
| **Browne, Sir T.** | *Religio Medici.* (Ed. by D. L. Roberts) | | 1898 |
| **Bryant, T.** | *On Ovariotomy* | (8) | 1867 |
| ,, | *The Practice of Surgery* | (4) | 1872 |
| **Bucknill, J. C.** | *Notes on Asylums for the Insane in America* | (6) | 1876 |
| **Burnett, C. M.** | *Insanity tested by Science* | (8) | 1848 |
| **Cabanis, P. J. G.** | *Rapports du Physique et du Moral de l'Homme.* 2 vols. | (4) 4e Éd. | 1824 |
| **Cabot, R. C.** | *Clinical Examination of the Blood* | 2nd Ed. | 1897 |
| **Campbell, H.** | *Respiratory Exercises* | | 1898 |
| *Catalogue of the Library of the Ophthalmological Society of the United Kingdom* | | (8) 2nd Ed. | 1893 |
| *Chambers's Biographical Dictionary* | | | 1897 |
| **Charcot, J. M.** | *Clinical Lectures on Senile and Chronic Diseases.* (Tr. by W. S. Tuke) | (6) | 1881 |
| **Chavasse,** [P.] | *Advice to a Mother.* 15th Ed. (Ed. by G. Carpenter) | | [1898] |
| ,, | *Advice to a Wife.* 14th Ed. (Ed. by F. Barnes) | | [1898] |
| **Chelius, M. J.** | *Handbuch der Chirurgie.* 2 bd. | (8) Dritte Auflage | 1830 |
| **Christie, J.** [Ed.] | *The Medical Institutions of Glasgow* | (8) | 1888 |
| **Christison, R.** | *A Treatise on Poisons* | (6) 4th Ed. | 1845 |
| ,, | *Dispensatory* | (6) 2nd Ed. | 1848 |
| **Clark, Sir J.** | *The Sanative Influence of Climate* | (8) 3rd Ed. | 1841 |

**Colbatch, J.** ... *Novum Lumen Chirurgicum; or a New Light of Chirurgery* ... (4) 4th Ed. 1699

**Coles, A. C.** ... *The Diseases of the Blood* ... 1898

**Coley, J. M.** ... *Diseases of Children* ... (6) 1846

**Combe, A.** ... *Physiology applied to the Preservation of Health* (4) 3rd Ed. 1835

**Cooke, W.** ... *Mind and the Emotions* ... (1) 1839

**Cooper, B. B.** ... *Hunterian Oration* ... (1) 1853

**Councilman, F. B. Mallory and J. H. Wright, W. T.** *Epidemic Cerebro-Spinal Meningitis and its Relation to other Forms of Meningitis* ... 1898

**Craigie, D.** ... *Practice of Physic.* 2 vols. ... (6) 1837–40

**Cullen, W.** ... *First Lines of the Practice of Physic.* 2 vols. (Ed. by P. Reid) ... (6) 1816

**Culpeper, [N.]** ... *English Physician and British Herbal.* (Ed. by Dr. Dickenson) ... (4) 1814

*Cyclopædia of Anatomy and Physiology, The.* (Ed. by R. B. Todd) 4 vols. (6) 1836–52

*Cyclopædia of Practical Medicine, The.* (Ed. by J. Forbes, A. Tweedie, and J. Conolly) Vols. I.–III. ... (6) 1833–34

**Davies, D.** ... *Report on a Localised Outbreak of Typhoid Fever in Bristol,* 1878 ... (8) 1879

**Demours, A. P.** ... *Les Maladies des Yeux* ... (8) 1821

**Dick, R.** ... *On the Derangements of the Digestive Organs* ... (4) 1840

**Dobson, N. C.** ... *Selections from Ten Years' Surgical Work* ... (6) 1885

**D'Orbigny, A.** ... *L'Homme Américain.* Tome I. ... (8) 1839

**Dowie, J.** ... *The Foot and its Covering* ... (6) 1861

**Druitt, R.** ... *The Surgeon's Vade-Mecum* ... (6) 5th Ed. 1851

**Ellis, G. V.** ... *Demonstrations of Anatomy* ... (4) 3th Ed. 1852

**Fayrer, Sir J.** ... *Sir James Ranald Martin* ... 1897

**Fell, J. W.** ... *A Treatise on Cancer* ... (8) 1857

**Fergusson, W.** ... *Notes and Recollections of a Professional Life.* (Ed. by J. Fergusson) ... (6) 1846

*Formulaire de la Faculté de Médecine de Vienne* ... 10e Éd. 1891

**Foville, [A.]** ... *Traité complet de l'Anatomie, de la Physiologie et de la Pathologie du Système nerveux cérébro-spinal* (8) 1844

**Fowler and R. J. Godlee, J. K.** *The Diseases of the Lungs* ... 1898

**Frankland, P. and Mrs. P.** *Pasteur* ... 1898

**French, Rev. A. J.** *The Life of John Birchenall* ... (4) [1881]

**Gadd, H. W.** ... *A Synopsis of the British Pharmacopœia,* 1898 ... 1898

**Gairdner, W. T.** ... *Clinical Medicine* ... (6) 1862

**Gant, F. J.** ... *From Our Dead Selves* ... 2nd Ed. 1897

**Gardner, A. K.** ... *The Conjugal Relationships as to Health* ... (6) 1892

**Garrod, A. B.** ... *Materia Medica and Therapeutics.* 4th Ed. (Ed. by E. B. Baxter) ... (6) 1874

**Gavin, H.** ... *On Feigned and Fictitious Diseases* ... (6) 1843

**Gellhorn, G.** ... *Radical-Behandlung des Gebärmutter-Scheidenkrebses* ... 1898

**Gersuny, R.** ... *Doctor and Patient.* (Tr. by A. S. Levetus) ... 1898

**Gibert, C.-M.** ... *Maladies spéciales de la Peau* ... (8) 2e Éd. 1840

**Godlee, J. K. Fowler and R. J.** *The Diseases of the Lungs* ... 1889

**Good, J. M.** ... *Nosology* ... (8) 1817

**Gordon, Sir C. A.** *Recollections of Thirty-nine Years in the Army* ... 1898

**Graves, R. J.** ... *Clinical Medicine.* (Ed. by Dr. Neligan). Vol. II. (6) 2nd Ed. 1884
**Greene, R.** ... *The Schott Treatment for Chronic Heart Diseases.* 2nd Ed. 1898
**Gregory, J.** ... *Conspectus Medicinæ Theoreticæ.* Translated (4) 1823
,, ... ,, ,, Translated (6) [2nd Ed.] 1844
**Gregory, O.** ... *Memoirs of John Mason Good* ... ... ... ... (1) 1832
**Gumprecht, F.** ... *Die Technik der speziellen Therapie* ... ... ... ... 1898
**Guthrie, G. J.** ... *Commentaries on the Surgery of War* ... (6) 5th Ed. 1853
**Hayward, J. R. S.** *Electro-Therapeia* ... ... ... ... ... ... ... (4) 1872
**Herman, G. E.** ... *Diseases of Women* ... ... ... ... ... ... ... 1898
**Hey, W.** ... *Practical Observations in Surgery* ... ... (6) 3rd Ed. 1814
**Hodgkin, T.** ... *De Absorbendi Functione* ... ... ... ... ... ... (6) 1823
,, ... *Morbid Anatomy of the Serous and Mucous Membranes.* Vols. I., II. Part I. ... ... (8) 1836-40
**Hooper, R.** ... *The Physician's Vade-Mecum* (6) 1809; (4) 5th Ed. (Ed. by W. A. Guy) 1856
**Husband, H. A.** ... *The Student's Handbook of Forensic Medicine* (4) 4th Ed. 1883
**Jacob, E. H.** ... *Ventilation and Warming* ... ... ... ... ... (6) 1894
**Jacobi, A.** .. *Therapeutics of Infancy and Childhood* ... 2nd Ed. 1898
**Jayne, H.** ... *Mammalian Anatomy.* Part I.—*The Skeleton of the Cat* ... ... ... ... ... ... ... ... ... ... 1898
**Johnstone, J.** ... *An Harveian Oration and other Remains* ... ... (4) 1837
*Katalog over det Medicinske Selskabs Bibliotek* ... ... ... ... ... ... ... 1898
**Kelly, H. A.** ... *Operative Gynecology.* Vol. I. ... ... ... ... ... ... 1898
**L.** ... *Parallele entre le Magnétisme animal, &c., appliqués aux Maladies rebelles* ... ... ... ... ... ... (4) 1785
**Lenné, A.** ... *Wesen, Ursache und Behandlung der Zuckerkrankheit* 1898
*Leprosy, Prize Essays on.* Second Series. ... ... ... ... ... ... ... ... 1897
**Lister, J.** ... *Observations on Ligature of Arteries on the Antiseptic System* ... ... ... ... ... (6) [2nd Ed.] 1869-70
**Macfarlane, J.** ... *Library Administration* ... ... ... ... ... ... ... ... 1898
**Mackenrodt, A.** ... *Das Studium der Frauenheilkunde* ... ... ... ... 1898
**Mackenzie, W.** ... *Diseases of the Eye* ... ... ... ... ... ... (8) 2nd Ed. 1835
**Magendie, [F.]** ... *Formulary for the Preparation and Employment of several New Remedies.* (Tr. from 6th Ed. by J. Houlton) ... ... ... ... ... ... ... ... (8) 1828
**Mallory and J. H. Wright, F.B.** *Pathological Technique* ... ... ... ... 1898
**Mallory, and J. H. Wright, W. T. Councilman, F. B.** *Epidemic Cerebro-Spinal Meningitis and its Relation to other Forms of Meningitis* ... ... ... ... ... ... 1898
**Mann, J. D....** ... *Forensic Medicine and Toxicology* ... ... ... 2nd Ed. 1898
**Markwick, A.** ... *On the Urine* ... ... ... ... ... ... ... ... ... (6) 1847
**Martin, J. R.** ... *The Influence of Tropical Climates on European Constitutions* ... ... ... .. ... (6) New Ed. 1856
**Mead, R.** ... ... *Medical Works* ... ... ... ... ... ... (6) New Ed. 1785
**Middlemore, R.** ... *Diseases of the Eye.* 2 vols. ... ... ... ... (8) 1835
**Mitchell, S. W.** ... *Fat and Blood* ... ... ... ... ... ... 7th Ed. 1898
**Monin, E.** ... ... *Hunyadi János* ... ... ... ... ... ... ... ... 1898
**Monsarrat, K. W.** *Surgical Technics* ... ... ... ... ... ... ... ... 1898
**Moore, J. E.** ... *Orthopedic Surgery* ... ... ... ... ... ... ... ... 1898
**Morgagni, J. B....** *The Seats and Causes of Diseases.* 2 vols. (Abridged by W. Cooke) ... ... ... ... ... ... ... (8) 1822

| | | |
|---|---|---|
| **Morgan, J.** ... ... | *The Dangers of Chloroform and the Safety and Efficiency of Ether* ... ... ... ... ... ... (4) | 1872 |
| **Morris, M.** ... ... | *Ringworm* ... ... ... ... ... ... ... ... ... ... ... | 1898 |
| **Morton, E.** ... ... | *Remarks on the Subject of Lactation* ... ... ... (8) | 1831 |
| **Moullin, C. M.** ... | *The Treatment of Sarcoma and Carcinoma by Injections of Mixed Toxins* ... ... ... ... ... ... ... ... | 1898 |
| ,, ,, ... | *Inflammation of the Bladder and Urinary Fever* ... ... | 1898 |
| **Moure, E. J.** ... | *Sur le Traitement des Sinusites (Maxillaire excepté)* ... | 1898 |
| **Newsholme, A.** ... | *Epidemic Diphtheria* ... ... ... ... ... ... ... ... | 1898 |
| *Nomenclature of Diseases, The* ... ... ... ... ... ... ... ... (6) 3rd Ed. | | 1896 |
| **Ödmansson, E.** ... | *Till Läran om Syphilis Congenita* ... ... ... ... | 1898 |
| **Paget, J.** ... | *Records of Harvey* ... ... ... ... ... ... ... ... (4) | 1846 |
| *Pamphlets.* 6 vols. | ... ... ... ... ... ... ... ... ... ... ... ... ... (6) | [v.y.] |
| **Paris, J. A.** ... | *A Treatise on Diet* ... ... ... ... (4) 1826, 2nd Ed. | 1827 |
| **Pattison, J.** ... | *Cancer* ... ... ... ... ... ... ... ... ... ... ... (4) | 1865 |
| **Peddie, A.** ... | *Dr. John Brown: His Life and Work; with Narrative Sketches of Syme* ... ... ... ... ... ... (6) | 1890 |
| **Pedley, R. D.** ... | *The Diseases of Children's Teeth* ... ... ... ... ... | [1895] |
| *Pharmacopœia, The British* ... ... ... ... ... ... ... ... ... ... ... | | 1898 |
| ,, ,, | *Additions to*, 1867 ... ... ... ... ... (6) | 1874 |
| **Philip, A. P. W.** | *Febrile Diseases.* Vol. I. ... ... (8) 3rd Ed. | 1813 |
| **Phillips, B.** ... | *A Treatise on the Urethra* ... ... ... ... .. (8) | 1832 |
| **Phillips, R.** ... | *Translation of the Pharmacopœia of the Royal College of Physicians*, 1851 ... ... ... ... ... (4) | 1851 |
| **Pickford, J. H.** ... | *Hygiene* ... ... ... ... ... ... ... ... ... (6) | 1858 |
| **Pott, P.** ... | *Chirurgical Works.* New Ed. (Ed. by Sir J. Earle) 3 vols. ... ... ... ... ... ... ... (6) | 1808 |
| **Prichard, A.** ... | *Ten Years of Operative Surgery in the Provinces.* Part II. ... ... ... ... ... ... ... (8) | 1863 |
| ,, | *Some Incidents in General Practice* ... ... ... ... | 1898 |
| **Prichard, J. C.** ... | *On the Relations of Ethnology to other Branches of Knowledge* ... ... ... ... ... ... ... (8) | 1847 |
| **Quain, J.** ... | *Elements of Anatomy.* 5th Ed. (Ed. by R. Quain and W. Sharpey) 2 vols. ... ... ... (6) | 1848 |
| **Ramsbotham, J.** ... | *Practical Observations in Midwifery.* 2 parts (6) | 1821-32 |
| **Roberts, D. L.** ... | *The Practice of Midwifery* ... ... ... 4th Ed. | 1896 |
| **Roberts-Jones, M.** | *Handbook on the Workmen's Compensation Act,* 1897 ... ... ... ... ... ... ... ... 5th Ed. | 1898 |
| **Robertson, A.** ... | *Lectures, &c.* ... ... ... ... ... ... ... ... ... (6) | 1862 |
| **Roger, [J.-B.-P.] Barth et H.** | *Traité pratique d'Auscultation* ... (8) | 1841 |
| **Ryan, W. B.** ... | *Infanticide* ... ... ... ... ... ... ... ... ... (4) | 1862 |
| *Sad Tones for Sick Times* ... ... ... ... ... ... ... ... ... ... (4) | | 1870 |
| **Schenk, L.** ... | *The Determination of Sex* ... ... ... ... ... ... | 1898 |
| **Schönlein, J. L.** ... | *Allgemeine und specielle Pathologie und Therapie.* 4 bd. (8) Zweite Auflage | 1832 |
| *Selecta è Præscriptis* ... ... ... ... ... ... ... ... ... (6) 11th Ed. | | 1851 |
| **Senn, N.** ... | *Tuberculosis of the Genito-Urinary Organs* ... ... ... | 1898 |
| *Sermons for the Jubilee of Queen Victoria* ... ... ... ... ... ... (4) | | 1887 |
| **Sewall, T.** ... | *An Examination of Phrenology* ... ... (8) 2nd Ed. | 1839 |
| **Shaffer, N. M.** ... | *Brief Essays on Orthopædic Surgery* ... ... ... ... | 1898 |
| **Shaw-Mackenzie, J. A.** | *Maternal Syphilis* ... ... ... ... ... ... ... | 1898 |

| | | | |
|---|---|---|---|
| Simpson, J. Y. | *Obstetric Works.* (Ed. by W. O. Priestley and H. R. Storer) 2 vols. | (6) | 1855-56 |
| ,, | *Physicians and Physic* | (6) | 1856 |
| ,, | *Acupressure* | (6) | 1864 |
| Skene, A. J. C. | *Diseases of Women* | 3rd Ed. | 1898 |
| Smith, A. | *Fever and Cholera* | (6) | 1873 |
| Smith, S. | *The Philosophy of Health.* 2 vols. | (4) | 1835-37 |
| Spencer, W. G. | *Outlines of Practical Surgery* | | 1898 |
| Squire, P. | *Companion to the British Pharmacopœia* | (6) 11th Ed. | 1877 |
| Stevenson, J. | *Weakness of Sight* | (8) 2nd Ed. | 1813 |
| Stewart, D. | *Philosophy of the Human Mind* | (1) 2nd Ed. | 1802 |
| Stewart, R. W. | *Diseases of the Male Urethra* | | 1896 |
| Stuart, Elma | *What mnst I do to get well? and How can I keep so?* | (6) 5th Ed. | 1892 |
| Syme, J. | *Contributions to the Pathology and Practice of Surgery* | (6) | 1848 |
| ,, | *On Stricture of the Urethra, &c.* | (6) 2nd Ed. | 1855 |
| Symonds, J. A. | *Ten Years: An Inaugural Lecture delivered at the Bristol Institution,* 1861 | (6) | 1861 |
| Taylor, A. S. | *Medical Jurisprudence* | (6) 3rd Ed. | 1849 |
| ,, | *The Principles and Practice of Medical Jurisprudence* | (6) | 1865 |
| Teale, T. P. | *Dangers to Health* | (6) 2nd Ed. | 1879 |
| Thayer, W. S. | *Lectures on the Malarial Fevers* | | 1897 |
| Thompson, C. J. S. | *Pharmacy and Dispensing for Nurses* | | 1898 |
| Thornton, J. H. | *Memories of Seven Campaigns* | | 1895 |
| Thresh, J. C. | *Water and Water Supplies* | 2nd Ed. | 1896 |
| Tilke, S. W. | *Nature and Treatment of Disease* | (4) 5th Ed. | 1844 |
| Walker, A. | *Beauty in Woman* | (6) 5th Ed. | 1892 |
| Waller, A. D. | *Exercises in Practical Physiology.* Parts I., III. | | 1897 |
| ,, | *Lectures on Physiology.* 1st Series.—*On Animal Electricity* | | 1897 |
| Warner, F. | *The Study of Children* | | 1898 |
| Waters, A. T. H. | *The Anatomy of the Human Lung* | (8) | 1860 |
| Weber, W. und E. | *Mechanik der menschlichen Gehwerkzeuge* | (8) | 1836 |
| Wells, T. S. | *Surgery, Past, Present and Future; and Excessive Mortality after Operations* | (6) | 1877 |
| West, C. | *Diseases of Women* | (6) 2nd Ed. | 1858 |
| ,, | *The Diseases of Infancy and Childhood* | (6) 5th Ed. | 1865 |
| White, W. | *Observations on the Contracted Intestinum Rectum* | (8) | 1812 |
| Wilde, P. | *The Elimination of Medical Sectarianism* | (4) | 1887 |
| ,, | *Therapeutic Progress and its Obstacles* | (4) | 1888 |
| Williams, C. J. B. | *Principles of Medicine* | (6) 2nd Ed. | 1848 |
| Williams, P. W. | *Diseases of the Upper Respiratory Tract* | 3rd Ed. | 1898 |
| Williamson, R. T. | *Diabetes Mellitus* | | 1898 |
| Wood, A. | *Homœopathy Unmasked* | (6) | 1844 |
| Wood, G. S. Woodhead and G. E. C. | *An Inquiry into the Relative Efficiency of Water Filters in the Prevention of Infective Disease* | | 1898 |
| Woodhead and G. E. C. Wood, G. S. | *An Inquiry into the Relative Efficiency of Water Filters in the Prevention of Infective Disease* | | 1898 |

**Wright, F. B. Mallory and J. H.** *Pathological Technique* ... ... ... ... 1898

**Wright, W. T. Councilman, F. B. Mallory, and J. H.** *Epidemic Cerebro-Spinal Meningitis and its Relation to other Forms of Meningitis* ... ... ... ... ... ... ... ... 1898

**Yearsley, J.** ... *A Treatise on the Enlarged Tonsil and Elongated Uvula* ... ... ... ... ... ... ... (4) 3rd Ed. 1848

---

### TRANSACTIONS, REPORTS, JOURNALS, &c.

Alienist and Neurologist, The... ... ... ... ... ... ... ... Vol. XVIII. 1897
American Journal of Obstetrics, The ... ... ... ... ... ... (9) Vol. XV. 1882
American Journal of Ophthalmology, The... ... ... ... ... Vol. XIV. 1897
American Laryngological Association, Transactions of the, 1897 ... ... 1898
American Year-Book of Medicine and Surgery, The ... ... ... ... 1898
Annalen der Irren-heilanstalt in Siegburg ... ... ... ... (8) Band I. 1837
Annales médico-psychologiques ... ... ... ... .. (8) Tomes III.-XI. 1844-48
Annual and Analytical Cyclopædia of Practical Medicine Vol. I. 1898
Archives cliniques de Bordeaux ... ... ... ... ... ... ... Tome VI. 1897
Archives of Surgery ... ... ... ... ... ... ... ... ... ... ... Vol. VIII. 1897
Australasian Medical Gazette, The ... ... ... ... ... ... Vol. XVI. 1897
Bäder-Almanach ... ... ... ... ... ... ... ... ... ... ... (10) [1898]
Boston Medical and Surgical Journal, The... ... ... Vol. CXXXVII. 1897
Bristol Medico-Chirurgical Journal, The ... ... ... ... Vol. XV. 1897
Bristol Port Sanitary District—Report for 1897... ... ... ... ... ... 1898
British Institute of Preventive Medicine, Transactions of the 1st Ser. 1897
Bulletin de l'Académie de Médecine. 3e Sér. Tomes XXXVII., XXXVIII. 1897
Bulletin de l'Académie royale de Médecine de Belgique. 4e Sér. Tome XI. 1897
Canadian Practitioner, The ... ... ... ... ... ... ... ... Vol. XXII. 1897
Centralblatt für klinische Medicin... ... ... ... ... ... ... ... 1881-92
Chrono-Thermalist ... ... ... ... ... ... ... ... ... ... ... ... (5) 1850
Clinical Journal, The ... ... ... ... ... ... ... ... ... Vol. XI. 1898
Clinical Society of London, An Index to Vols. I.—XXX. of the Transactions of the ... ... ... ... ... ... ... ... ... ... ... ... ... ... 1898
Dermatological Society of Great Britain and Ireland, Transactions of the ... ... ... ... ... ... ... ... ... ... ... ... ... Vol. III. 1897
Dictionary of Bathing Places, &c., Bradshaw's .. ... ... ... ... ... 1898
Directory, Bristol and Clifton ... ... .. ... ... ... ... ... (6) 1886
Epidemiological Society of London, Transactions of the. N.S. Vol. XVI. 1897
Fishponds Private Lunatic Asylum Inquiry ... ... ... ... ... (6) 1848
Gazette des Hôpitaux de Toulouse ... ... ... ... ... ... 1894, 1895
Hospital, The ... ... ... ... ... ... ... ... ... ... ... ... Vol. XXIII. 1898
Hospitals—
Johns Hopkins Hospital Reports, The .. Vol. VII., Nos. 1-2 1898
Middlesex Hospital, The. Registrars' Reports for (2) 1876-79; (7) 1895; 1896
Ophthalmic Hospital Reports ... ... ... (8) Vols. I.—III. 1857-61
Rotunda Hospital—Clinical Report for 1896-97 ... ... ... ... 1898
Intercolonial Medical Journal of Australasia ... ... ... ... Vol. II. 1897
International Journal of Surgery, The... ... (9) Vols. VI.—VIII. 1893-95
International Medical Magazine ... ... ... ... ... ... Vol. VI. 1897
Johns Hopkins Hospital Bulletin, The... ... ... ... ... Vol. VIII. 1897
Journal of Experimental Medicine, The ... ... ... ... Vol. II. 1897
Journal of the American Medical Association, The. Vols. (3) I., II., 1883-84; III., 1884, XXI., 1893, XXIII., 1894, XXIX. 1897
Leistungen und Fortschritte der Medizin in Deutschland, Die. (8) Band I. 1833
Library, The ... ... ... ... ... ... ... ... ... ... ... ... Vol. IX. 1897
Liverpool Medico-Chirurgical Journal, The ... ... .. Vol. XVII. 1897
Medical and Surgical Reporter, The. Vols. (9) XXXII., XXXIII., 1875-76, XXXVII.—XLIX., 1877-83; LXXVII. 1897
Medical Annual, The ... ... ... ... ... ... ... ... ... ... 1898

Medical News and Abstract, The ... (9) Vols. XXXVIII., XXXIX. 1880-81
Medical News and Library, The ... (9) Vols. XXVII., 1869, XXIX. 1871
Medicinische Bibliographie ... ... ... ... ... ... ... ... ... 1883, 1884
Montreal Medical Journal, The ... ... ... ... ... ... Vol. XXVI. [1897]
Nord médical, Le ... ... ... ... ... ... ... ... ... Tome III. 1897
Northumberland and Durham Medical Journal... ... ... ... ... ... 1897
Occidental Medical Times ... ... ... ... ... ... ... Vol. XI. 1897
Ohio State Medical Society, Transactions of the ... ... ... ... ... 1897
Philadelphia, Transactions of the College of Physicians of. 3rd Ser. Vol. XIX. 1897
Quarantine, Second Report on—Yellow Fever. With Appendices (8) 1852
Railway Surgeon, The ... ... ... ... ... ... (9) Vols. I., II. 1894-96
St. Petersburger medicinische Wochenschrift ... ... ... ... ... ... 1897
Treatment ... ... ... ... ... ... ... ... ... ... ... ... Vol. I. 1898
Zeitschrift für Krankenpflege ... ... ... ... ... ... ... ... ... ... 1897

### Local Medical Notes.

### BRISTOL.

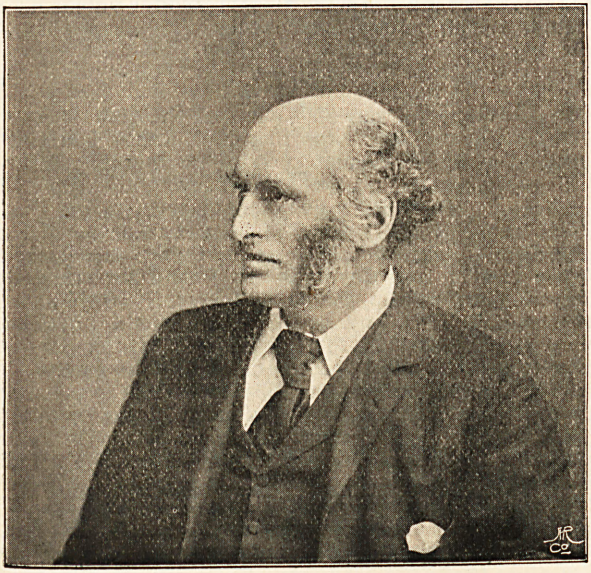

Obituary.—By the death of Dr. Henry Marshall, on Sunday, the 24th of April, after a few days' illness, the profession and public of Bristol have suffered a great loss. A son of a former Vicar of Christ Church, Clifton, he received the elements of his medical education at the Bristol Medical School, completing it at the University of Edinburgh, where he took his M.D. He was also F.R.S.E. There is little doubt that the magnetic influence of Syme, which so dominated the Edinburgh School, induced Dr. Marshall, on his starting practice, to adopt the career of a surgeon, and it was in this capacity that for ten years he was attached to the Bristol General Hospital. From 1863 to 1872 he lectured on Forensic Medicine at the School. His tastes and inclinations, however, led him to resign this appointment and practise as a physician, for which his cultured manner eminently fitted him; as such, he had for many years a very large practice in Clifton. He was at one time Secretary of the Bath and Bristol Branch of the British Medical Association, and afterwards became its President. It was while he held the post of Secretary that the Association paid its second visit to Bristol. At one time he took part frequently in the discussions at the meetings, but in later years he seldom came, and still less frequently spoke. Dr. Marshall had many interests outside his profession. He was a man of unbounded vigour and spirit, and his 65 years sat lightly on him. He was an energetic churchman, and for some time he had been President of the local branch of the English Church Union. He was a fluent forcible speaker, and had the gift of expressing his thoughts in well-chosen words. His death will be felt by numerous friends and patients who admired the strong, sincere and striking worth of the man, and the kindly courteousness with which his name will ever be associated.

Under the presidency of the Dean of Bristol, a meeting has been held at which it was resolved to establish at the General Hospital a Memorial to Dr. Marshall and to ask for permission to place a tablet in the Cathedral. Subscriptions for the "Marshall Memorial Fund" should be sent to Messrs. Stuckeys' Banking Company, Limited.

Greig Smith Memorial.—On May 5th the High Sheriff (Mr. F. Richardson Cross), in the presence of a large number of persons, unveiled the bronze bust of the late James Greig Smith which has been placed in the lobby of the Museum. Mr. J. W. Arrowsmith, the Chairman of the Memorial Committee, said that £520 had been collected, and that after mature consideration it had been determined to place a bust in the Museum, a medallion in the operating theatre of the Infirmary, and give the remainder to the Committee of that Institution to assist in the refitting of that room. The High Sheriff, in a few well-chosen words, referred to the great abilities of Mr. Greig Smith, and the severe loss the surgical world and the public had suffered by his death at a comparatively early age.

### EXETER.

Obituary.—We regret having to record the loss of one of our district Editors, Dr. Arthur George Blomfield, whose death, at the age of 43, occurred at his residence, 34 West Southernhay, Exeter. He received his professional education at Aberdeen University and King's College, London. He became qualified in 1877, taking the L.S.A., and in 1878 the M.R.C.S. In 1882 he obtained the M.D. degree of the Aberdeen University. He was for three years house-surgeon to the West Norfolk and Lynn Hospital, and for six and a half years house-surgeon at the Devon and Exeter Hospital. He resigned this post in 1887, and began practice as a physician. Dr. Blomfield was physician to the Devon and Exeter Hospital, the Exeter Dispensary, the West of England Institution for the Deaf and Dumb, and to the Exeter Lying-in Charity. He was a gentleman of extremely courteous manners. As a consultant, he maintained the highest traditions of the profession, and could always be relied upon to consider both the interests of the patient and of the medical man in attendance. His popularity with his poor patients was evidenced by the large numbers of them who attended his funeral.

Dr. R. V. Solly has kindly undertaken to act as our Editorial representative in his neighbourhood in succession to Dr. Blomfield.

### Congress for the Consideration of Tuberculosis.

Professor Nocard calls our attention to the fact that the Fourth Congress on this subject will be held in Paris from July 27th to August 2nd. Dr. L. H. Petit, 18 Rue du Pré-aux-Clercs, Paris, will be pleased to give information in detail concerning it.

### The Sanitary Institute.

The 17th Congress of the Sanitary Institute will begin at Birmingham on September 27th, with Sir Joseph Fayrer as President.

### SCRAPS.

### *PUBLICATIONS RECEIVED.*

From D. Appleton and Company, New York:

*Lectures on the Malarial Fevers.* By William Sydney Thayer, M.D. 1897.
*Essays on Orthopædic Surgery.* By Newton M. Shaffer, M.D. 1898.
*Diseases of Women.* By Alexander J. C. Skene, M.D., LL.D. Third Edition. 1898.

From J. W. Arrowsmith, Bristol:

*Some Incidents in General Practice.* By Augustin Prichard. 1898.

From Baillière, Tindall and Cox, London:

*Respiratory Exercises in the Treatment of Disease.* By Harry Campbell, M.D., B.S. 1898.
*From our Dead Selves.* By Frederick James Gant. Second Edition. 1897.
*Outlines of Practical Surgery.* By Walter G. Spencer, M.B., M.S. 1898.

From John Bale, Sons and Danielsson, Ltd., London:

*The Treatment of Sarcoma and Carcinoma by Injections of Mixed Toxins.* By C. Mansell Moullin, M.D. 1898.
*The Journal of Balneology and Climatology.* April, 1898.

From Friedr. Bayer & Co., Elberfeld:

*Clinical Excerpts.* January—May, 1898.

From Bennett Brothers, Ltd., Bristol:

*Bristol Port Sanitary District. Annual Report of the Medical Officers of Health and of the Chief Port Inspector of Nuisances for the Year 1897.* 1898.

From Adam and Charles Black, London:

*Diseases of the Heart.* By George William Balfour, M.D., LL.D., F.R.S.E. Third Edition. 1898.

From Boot's Pure Drug Co., Ltd., Nottingham:

*Review of the New British Pharmacopœia, 1898, with Notes of Alterations and Additions.*

From The British Medical Association, London:

*An Inquiry into the Relative Efficiency of Water Filters in the Prevention of Infective Disease.* By G. Sims Woodhead, M.D., F.R.S.E., and G. E. Cartwright Wood, M.D., B.Sc. 1898.

From California Medical College, San Francisco:

*California Medical Journal.* March—May, 1898.

From Cassell and Company, Limited, London:

*Pasteur.* By Percy Frankland, Ph.D., B.Sc., F.R.S., and Mrs. Percy Frankland. 1898.
*The South African Climate.* By William C. Scholtz, M.D. 1897.
*Diseases of Women.* By George Ernest Herman, M.B. 1898.
*Ringworm.* By Malcolm Morris. 1898.

From Church Missionary Society, London:

*Mercy and Truth.* April—June, 1898.

From J. & A. Churchill, London:

*Maternal Syphilis.* By John A. Shaw-Mackenzie, M.D. 1898.
*Chavasse's Advice to a Wife.* Fourteenth Edition. Revised by Fancourt Barnes, M.D., F.R.S.E. 1898.
*Chavasse's Advice to a Mother.* Fifteenth Edition. By George Carpenter, M.D. 1898.
*The Diseases of the Blood.* By Alfred C. Coles, M.D. 1898.

From Archibald Constable and Co., Westminster:

*Memories of Seven Campaigns.* By James Howard Thornton, C.B., M.B., B.A. 1895.

From Eduardo Dublan, Mexico:

*Boletin del Consejo superior de Salubridad.* Febrero 28, Marzo 31, Abril 30, 1898.

From The Dunlop Pneumatic Tyre Company, Limited, London:

*Dunlop News.* Spring, 1898.

From Dr. George M. Edebohls, New York:

*The Other Kidney in Contemplated Nephrectomy.* By George M. Edebohls, A.M., M.D. 1898. [Reprint.]

From Dr. Landon B. Edwards, Richmond, U.S.A.:

*Virginia Medical Semi-Monthly.* February 25—May 13, 1898.

From Evans, Gadd & Co., Exeter:

*B.P. Synopsis, 1898.* By H. Wippell Gadd. 1898.

From John Falconer, Dublin:

*Clinical Report of the Rotunda Hospital, for one year, November 1, 1896, to October 31, 1897.* 1898.

From Feret et Fils, Bordeaux:

*Sur le Traitement des Sinusites (Maxillaire excepté).* Par le Dr. E. J. Moure. 1898.
*Ueber die chirurgische Behandlung der Otitis media chronica sicca.* Von Dr. E. J. Moure. [n.d.] [Reprint.]

From Ferris & Company, Bristol:

*Pocket Notes on the New British Pharmacopœia.* 1898.

From Gustav Fischer, Jena:

*Die Technik der speziellen Therapie.* Von Dr. F. Gumprecht. 1898.

From Dr. L. Webster Fox, Philadelphia:

*Ophthalmia Neonatorum.* By L. Webster Fox, M.D. 1897. [Reprint.]

From Dr. George M. Gould, Philadelphia:

*Some Relations of Author, Publisher, Editor, and Profession.* By George M. Gould, M.D. 1897. [Reprint.]
*Child Fetiches.* By George M. Gould, M.D. 1898. [Reprint.]

From T. & W. Goulding, Bristol:

*Bristol Eye Dispensary. Annual Report for 1897.* 1898.

From Charles Griffin and Company, Limited, London:

*Forensic Medicine and Toxicology.* By J. Dixon Mann, M.D. Second Edition. 1898.

From Drs. P. Hartenberg & P. Valentin, Paris:

*Revue de Psychologie.* Fév.—Mai, 1898.

From Henry Hodder & Co., Ld., Bristol:

*Captol.* By Dr. P. J. Eichhoff. 1897. [Reprint.]

From Dr. Joseph H. Hunt, Brooklyn:

*Who Discovered Anæsthesia?* By Joseph H. Hunt, M.D. 1896. [Reprint.]

From Ingram Brothers, London:

*The English Illustrated Magazine.* May, 1898.

From A. D. Innes and Co., London:

*Sir Ranald Martin.* By Surgeon-General Sir Joseph Fayrer, Bart., K.C.S.I., LL.D., M.D., F.R.S. 1897.

From L'Institut de Bibliographie, Paris:

*Gazette médicale de Paris.* 12, 19, 26 Mars, 2, 9, 16, 23 Avril, 1898.

From The Johns Hopkins Press, Baltimore:

*The Johns Hopkins Hospital Reports.* Vol. VII., Nos. 1–2. *Report in Gynecology.* 1898.

From S. Karger, Berlin:

*Das Studium der Frauenheilkunde, ihre Begrenzung innerhalb der allgemeinen Medicin.* Von Dr. A. Mackenrodt. 1898.

*Ueber die Resultate der Radical-Behandlung des Gebärmutter-Scheidenkrebses mit dem Glüheisen* Von Dr. Georg Gellhorn. 1898.

*Wesen, Ursache und Behandlung der Zuckerkrankheit.* Von Dr. Albert Lenné. 1898.

From Henry Kimpton, London:

*Operative Gynecology.* By Howard A. Kelly, A.B., M.D. Vol. I. 1898.

From H. K. Lewis, London:

*Diseases of the Kidney and Urinary Organs.* By Professor Dr. Paul Fürbringer. Translated by W. H. Gilbert, M.D. Vol. II. 1898.
*Yellow Fever.* By Izett Anderson, M.D. 1898.
*Inflammation of the Bladder.* By C. Mansell Moullin, M.D. 1898.
*Middlesex Hospital Reports for 1896.* 1897.
*Aphasia and other Speech Defects.* By H. Charlton Bastian, M.A., M.D., F.R.S. 1898.

From J. B. Lippincott Company, London:

*Mammalian Anatomy.* By Horace Jayne, M.D., Ph.D. Part I.—*The Skeleton of the Cat.* 1898.
*Therapeutics of Infancy and Childhood.* By A. Jacobi, M.D. Second Edition. 1898.

From Longmans, Green, and Co., London:

*The Diseases of the Lungs.* By James Kingston Fowler, M.A., M.D., and Rickman John Godlee, M.S. 1898.
*Essentials of Experimental Physiology.* By T. G. Brodie, M.D. 1898.
*Tablets of Anatomy.* By Thomas Cooke, B.A., B.Sc., M.D., and F. G. Hamilton Cooke. Three Parts. [Eleventh Edition.] 1898.

From Alex. Macdougall, Glasgow:

*A Series of Specimens illustrative of Certain Congenital Affections of the Urinary Apparatus.* By L. R. Sutherland, M.B., and G. H. Edington, M.D., C.M. 1898. [Reprint.]

From Macmillan and Co., Limited, London:

*A System of Medicine.* By Many Writers. Edited by Thomas Clifford Allbutt, M.A., M.D., LL.D., F.R.S. Vol. V. 1898.

### PUBLICATIONS RECEIVED *(continued)*.

From The Macmillan Company, New York:
*The Study of Children.* By Francis Warner, M.D. 1898.

From P. A. Norstedt & Soner, Stockholm:
*Till Läran om Syphilis congenita.* Af Professor E. Ödmansson. 1898.

From Kegan Paul, Trench, Trübner & Co., Ltd., London:
*B. Bradshaw's Dictionary of Bathing Places, &c.* 1898.

From Young J. Pentland, Edinburgh:
*Diabetes Mellitus.* By R. T. Williamson, M.D. 1898.

From Pharmaceutical Journal Office, London (per T. Buxton & Co., Bristol):
*The British Pharmacopœia, 1898. Alterations, Additions, and Omissions. A Pocket Guide for Pharmacists and Students.* Sixth Edition. 1898.

From Pontieri & Velardi, Naples:

*La Ictalbina* (Knoll) *nella odierna terapia,* [G. Scognamiglio.] 1898. [Reprint.]

From The Rebman Publishing Co., Limited, London:
*Archives of the Roentgen Ray.* February, 1898.
*Pathological Technique.* By Frank Burr Mallory, A.M., M.D., and James Homer Wright, A.M., M.D. 1898.
*Orthopedic Surgery.* By James E. Moore, M.D. 1898.
*Tuberculosis of the Genito-Urinary Organs.* By N. Senn, M.D., Ph.D., LL.D. 1898.
*Genito-Urinary and Skin Diseases, including Syphilis.* Edited by L. Bolton Bangs, M.D., and W. A. Hardaway, A.M., M.D. One volume in two. 1898.

From Andreas Saxlehner, Budapest:
*Hunyadi János.* By Dr. E. Monin. 1898.

From The Scientific Press, Limited, London:
*Notes on Pharmacy and Dispensing for Nurses.* By C. J. S. Thompson. 1898.
*The Schott Treatment for Chronic Heart Diseases.* By Richard Greene. Second Edition. 1898.

From J. P. Segg & Co., London:
*The Diseases of Children's Teeth.* By R. Denison Pedley. [1895 ]

From J. W. Simpson, Derby:
*Derby Borough Asylum. Ninth Annual Report, 1897.* 1898.

From Swan Sonnenschein & Co., Ltd., London:
*Epidemic Diphtheria.* By Arthur Newsholme, M.D. 1898.
*Recollections of Thirty-Nine Years in the Army.* By Sir Charles Alexander Gordon, K.C.B. 1898.

From Spottiswoode & Co., London:
*The British Pharmacopœia.* 1898.

From Steenske Bogtrykkeri, Christiania:
*Katalog over det Mediciniske Selskabs Bibliotek.* 1898.

From Tipografia Fratelli Centenari, Rome:
*L'Organo-Terapia nelle Nefriti della Infanzia.* Per Prof. Luigi Concetti. 1898. [Reprint.]

From The Werner Company, London:
*The Determination of Sex.* By Dr. Leopold Schenk. 1898.

From Western Mail, Limited, Cardiff:
*Handbook on the Workmen's Compensation Act, 1897.* By M. Roberts-Jones. Fifth Edition. 1898.

From William Wood and Company, New York:
*Diseases of the Male Urethra.* By R. W. Stewart, M.D. 1896.

From John Wright & Co., Bristol
*Surgical Technics.* By K. W. Monsarrat, M.B. 1898.
*Rheumatoid Arthritis.* By Gilbert A. Bannatyne, M.D. Second Edition. 1898.
*Diseases of the Upper Respiratory Tract.* By P. Watson Williams, M.D. Third Edition. 1898.

From Wright & Potter Printing Co., Boston:
*Epidemic Cerebro-spinal Meningitis and its Relation to other forms of Meningitis.* By Dr. W. T. Councilman, Dr. F. B. Mallory, and Dr. J. H. Wright. 1898.

From K. J. Wyss, Berne:
*Illustrirte Rundschau der Medicinisch-chirurgischen Technik.* 15 Mai, 1898.

